# Wound-Induced Regeneration in Feather Follicles: A Stepwise Strategy to Regenerate Stem Cells

**DOI:** 10.3390/jdb13020010

**Published:** 2025-03-27

**Authors:** Ting-Xin Jiang, Ping Wu, Ang Li, Randall B. Widelitz, Cheng-Ming Chuong

**Affiliations:** 1 Department of Pathology, Keck School of Medicine, University of Southern California, Los Angeles, CA 90033, USA; tjiang@usc.edu (T.-X.J.); pingwu@usc.edu (P.W.); ang.li3@uta.edu (A.L.); widelitz@med.usc.edu (R.B.W.); 2 Department of Kinesiology, College of Nursing and Health Innovation, The University of Texas at Arlington, Arlington, TX 76019, USA

**Keywords:** skin appendages, dermal papilla, wounding, regeneration, stem cells, feather cycling, hair follicle

## Abstract

How to elicit and harness regeneration is a major issue in wound healing. Skin injury in most amniotes leads to repair rather than regeneration, except in hair and feathers. Feather follicles are unique organs that undergo physiological cyclic renewal, supported by a dynamic stem cell niche. During normal feather cycling, growth-phase proximal follicle collar bulge stem cells adopt a ring configuration. At the resting and initiation phases, these stem cells descend to the dermal papilla to form papillary ectoderm and ascend to the proximal follicle in a new growth phase. Plucking resting-phase feathers accelerates papillary ectoderm cell activation. Plucking growth-phase feathers depletes collar bulge stem cells; however, a blastema reforms the collar bulge stem cells, expressing KRT15, LGR6, Sox9, integrin-α6, and tenascin C. Removing the follicle base and dermal papilla prevents feather regeneration. Yet, transplanting an exogenous dermal papilla to the follicle base can induce re-epithelialization from the lower follicle sheath, followed by feather regeneration. Thus, there is a stepwise regenerative strategy using stem cells located in the collar bulge, papillary ectoderm, and de-differentiated lower follicle sheath to generate new feathers after different levels of injuries. This adaptable regenerative mechanism is based on the hierarchy of stem cell regenerative capacity and underscores the remarkable resilience of feather follicle regenerative abilities.

## 1. Introduction

How stem cells are regulated during tissue renewal or activated after wounding is a fundamental yet unsolved question. Following injury, human tissues typically undergo repair that covers wounds with epidermis and connective tissue but fails to restore complete functionality. In contrast, many animals exhibit more robust regenerative abilities, offering principles that may inspire strategies for human therapies. Animals capable of regeneration generate a wound blastema via dedifferentiation or activation of adult tissue stem cells [1,2,3]. This can be seen following the loss of limbs in some species. During limb bud development, limb bud progenitors are in the distal limb bud, under the apical epidermal ridge, while the proximal limb bud gradually differentiates into skeletal elements [4]. Eventually, all cells differentiate and there are no resident stem cells left in the adult limb. When the adult limb is amputated, a wound blastema emerges from cells of different lineages via de-differentiation, as seen in the axolotl [5]. Missing limb segments are then reformed in appropriate locations [6].

While adult humans and mammals have lost their regenerative abilities in many organ systems, the distal digit tip retains this ability [7,8]. Digit tip epithelial stem cells have dual functionality, enabling them to regenerate both the nail and surrounding epithelium [9]. While adult amphibians can regenerate their skin with full functionality following wounding [10], wounds often induce scarring in mammals.

When injuries occur on the integument, the body tries to rebuild the tissue architecture. Depending on the depth of wounding, different epithelial cells are activated to heal the wound [11]. In the most dramatic example, when a large full-thickness wound is created on the backs of mice, new hair placodes and dermal condensations re-emerge to rebuild new hair follicles in the process known as wound-induced hair neogenesis [12]. This full regeneration of skin appendages is also observed in reptile scales. While injuring parts of scales leads to a repair response, full new scale arrays can reform in the tail epidermis during tail regeneration [13]. These examples demonstrate the full regeneration of whole follicles after a piece of skin is lost. What happens when skin appendage follicles are partially wounded or removed in an experimental setting? In classical experiments, when a hair follicle base was transected, a transplanted dermal papilla (DP) could interact with the host bulge stem cells to establish a blastema for hair regeneration [14]. Feather follicle epidermal and dermal cells have also been partially dissected, and the ability to regenerate partial or full feathers in different inductive conditions has been evaluated [15].

Hair and feathers are unique in that they undergo physiological cyclic renewal and have robust regenerative abilities [16]. During hair regeneration, bulge stem cells remain for the next cycle, but lower regions of the follicles degenerate [17]. During telogen, some hair stem cells move down to the hair germ, which lies above the DP and is involved in inducing a new hair cycle [18]. Later, the hair germ and outer root sheath stem cells are able to dedifferentiate and reform bulge stem cells [19,20,21]. Despite a similarity in structure, hair and feather follicles result from convergent evolution. They share a proximal follicle structure housing long-term label-retaining stem cells (LRCs) and transient amplifying (TA) cells, with the distal filament differentiating first [22,23,24]. In feathers, TA cells are proliferative cells localized in the basal layer of the feather epidermis and the peripheral pulp. LRCs, which are slow-cycling and considered putative stem cells, are present in both epidermal and dermal layers [25,26]. Epidermal LRCs reside in the collar bulge, while dermal LRCs are found in the peripheral pulp, adjacent to their epidermal counterparts. During feather cycling, both epidermal and dermal LRCs co-migrate and descend to the follicle base during the resting phase [26].

Here we study how different feather follicle components respond to injury and what strategies are used to regenerate feathers. Feathers have played a critical role in helping dinosaurs and birds adapt to their environment. Hence, a robust regenerative strategy in feather follicles must have evolved early to ensure successful feather regeneration that enhanced the competitive survival of feathered dinosaurs [27] and Mesozoic birds [28,29,30]. Modern birds effectively regenerate feathers through seasonal molting, following injury, and after experimental recombination to form chimeric follicles [15,31]. This ability is due to the feather follicle architecture, which houses epithelial stem cells within a protective niche.

**Figure 1:**
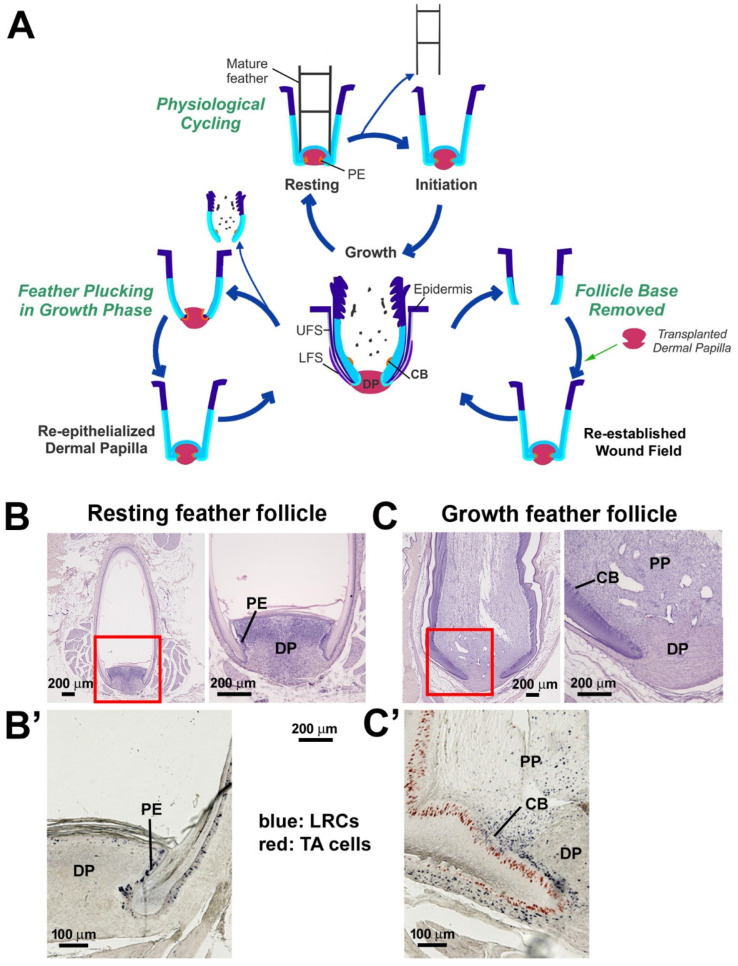
**Physiological and wound-induced feather regeneration.** (**A**) Three loops depict modes of feather regeneration under normal physiological cycling (middle loop), regeneration after plucking growth phase follicles (left lower loop), and reconstruction of chimeric follicles after DP transplantation (right lower loop). (**B**) H&E staining of a resting phase feather follicle. The mature feather shaft is fully keratinized. The DP is surrounded by PE. (**B’**) In the resting phase, LRCs (blue) are located in the PE. TA cells (red) are rarely detected. (**C**) H&E staining of a growth phase feather follicle. The red boxes in the left panels show the areas that are magnified in the right of panels (**B**,**C**). (**C’**) In the growth phase, LRCs (blue) are located in the CB, whereas TA cells (red) can be found in the basal epidermal cells. CB, collar bulge; DP, dermal papilla; LRC, label-retaining cells; PE, papillary ectoderm; LFS, lower follicle sheath; PP, pulp; TA cells, transient amplifying cells; UFS, upper follicle sheath.

Unlike hair, feathers do not cyclically destroy their lower follicles [32,33]. Instead, the overall follicle architecture is maintained (Figure 1A). In the growth phase (Figure 1C), stem cells are located in a ring configuration within the collar bulge [25], and LRCs are absent from the papillary ectoderm (PE) and lower follicle sheath under physiological cycling (Figure 1C’, blue-colored). In the resting phase (Figure 1B), this stem cell ring descends to the PE region, located at the flexure point of the follicle sheath and the collar epithelium (Figure 1B’, blue-colored). In addition, some LRCs are scattered in the lower follicle sheath. Cell proliferation is active in the collar and follicle sheath of the growth phase follicle (Figure 1C’, red-colored). In both cases, cells flow distally and differentiate [25]. As proliferation tapers in the resting follicle, LRCs descend to the PE region, waiting to be re-activated in the next feather cycle [26]. Thus, in normal regenerative cycling, feather stem cells achieve homeostasis by shifting back and forth between two locations within the progenitor niche.

In addition to physiological cycling, feathers can be lost due to injury and then regenerate. However, a systemic study evaluating how the stem and TA cells cope with various follicle injuries has not been undertaken. In this paper, we focus on feather regeneration after different types of injuries to feathers or their follicles. The objective here is to study feather follicle progenitor plasticity and to compare our findings with other organs that regenerate, to identify the essential requirement(s) for establishing a wound blastema. We compare two plucking-induced regeneration conditions in which feathers can fully regenerate (Figure 1A). One is feather plucking from the resting phase, which uses the activation of resident stem cells in the PE region. The other is plucking from the growth phase, which requires re-epithelialization from the lower dermal sheath to establish the blastema required for feather regeneration. We then tested the third condition by removing the follicle base. Under this condition, regeneration failed unless a DP was transplanted to re-establish the blastema for feather regeneration.

## 2. Materials and Methods

### 2.1. Plucking and Surgery of Feather Follicles

White leghorn chickens are hatched from eggs that were purchased from Charles River Laboratories. One-year-old adult male chickens were used in the experiments. All the animals used in this study were processed following an approved protocol (11903) of the Institutional Animal Care and Use Committees of the University of Southern California (Los Angeles, CA). For anesthesia, chickens were injected with 10 mg per kg (body weight) of ketamine and xylazine (9:1).

For regenerating feather collection, dorsal contour feathers were plucked at either the growth or resting phases feather cycle. Feather follicles are collected at 0 h (immediately after plucking), 1 day, 4 days, and 8 days. Five feather follicles in each condition were prepared.

In transplantation experiments, the DP plus the lower follicle from the host feather follicle were removed (N = 5). For the donor, the DP was removed from a different feather follicle in the same chicken (N = 5). To remove the PE, donor DP is incubated in 2× CMF for 20–40 min at 4 °C. Then, the epithelia (PE) are carefully removed with watchmaker’s forceps. DP with or without PE was transplanted to the host follicle (N = 5 for each). The host follicle without dermal papilla and lower follicle was used as a control (N = 5). Five experiments were performed in each condition. Feather follicles were collected at designated days after transplantation.

### 2.2. Hematoxylin and Eosin (H&E) and Immunostaining

Feather follicles were fixed in 4% paraformaldehyde at 4 °C overnight, and 7 μm longitudinal paraffin sections were prepared according to standard procedures. Hematoxylin and Eosin (H&E) and immunostaining were followed by procedures previously described by [26]. For immunostaining, sections were dewaxed with xylene twice (10 min each) and then rehydrated with an ethanol series. After washing with PBS twice (5 min each), slides were treated with 3% H_2_O_2_ in methanol (10 min) and washed with PBS 4 times (5 min each). Sections were blocked with Zeller solution (10 mM Tris-HCl (Sigma-Aldrich, St. Louis, MO, USA), 100 mM MgCl_2_ (Sigma-Aldrich, St. Louis, MO, USA), 5% fetal bovine serum (VWR), 1% BSA (Sigma-Aldrich, St. Louis, MO, USA), 0.5% Tween-20 (Sigma-Aldrich, St. Louis, MO, USA), pH 7.4) for 30 min. Primary antibodies were applied overnight. After washing in PBS 3 times (5 min each), biotinylated goat anti-mouse IgG or biotinylated goat anti-rabbit IgG (Vector Labs, Newark, CA, USA, 1:200) was used as a secondary antibody (1 h). Streptavidin (Jackson ImmunoResearch, West Grove, PA, USA, 1:200) was used as a tertiary antibody (1 h). AEC Substrate Kit, Peroxidase (HRP) (Vector Labs, Newark, CA, USA) was used to develop the red color.

We used the following primary antibodies: β-catenin (Sigma-Aldrich, St. Louis, MO, USA, 1:200), integrin-α6 (Developmental Studies Hybridoma Bank, Iowa City, IA, USA, 1:10), and tenascin C, which was from the Chuong lab [34] (1:200).

### 2.3. Cell Proliferation Study

BrdU was used to locate long-term label-retaining cells. Adult chickens were fed BrdU (Sigma-Aldrich, St. Louis, MO, USA, 0.1 mg/mL) in their drinking water for 1 week and then ‘chased’ for 10 days. Feathers were plucked from follicles at the growth or resting phase as described above (N = 5 for each condition). Unplucked follicles were used as controls. To double label LRCs and TA cells in a feather follicle, we fed chickens IdU (Sigma-Aldrich, St. Louis, MO, USA, 0.1 mg/ ml) for 1 week. After a 10-day chase, CldU (Sigma-Aldrich, St. Louis, MO, USA, 2 mg/kg) was injected, and feather follicles (N = 10) were collected 3 h later.

BrdU was detected by adding mouse anti-BrdU (BD Biosciences, San Diego, CA, USA, 1:200). Biotinylated goat anti-mouse IgG (Vector Labs, Newark, CA, USA, 1:200) and streptavidin (Jackson ImmunoResearch, West Grove, PA, USA, 1:200) were used as secondary and tertiary antibodies; AEC Substrate Kit, Peroxidase (HRP) (Vector Labs, Newark, CA, USA) was used to develop the red color.

For IdU/CldU double staining, sections were treated with 0.01 M citrate buffer (pH 6.0) by microwaving for 6 min. CldU was detected using a rat anti-BrdU antibody (BU 1/75; Abcam, Fremont, CA, USA, 1:200); biotinylated goat anti-rat IgG (Vector Labs, 1:200) and streptavidin (Jackson ImmunoResearch, West Grove, PA, USA, 1:200) were used as secondary and tertiary antibodies; AEC Substrate Kit, Peroxidase (HRP) (Vector Labs, Newark, CA, USA) was used to develop the red color. IdU was detected by mouse anti-BrdU (347580; BD Biosciences, San Diego, CA, USA, 1:200); anti-mouse AP (Abcam, Fremont, CA, USA, 1:200) was used as a secondary antibody; NBT/BCIP (Promega, Madison, WI, USA) was used to visualize positive staining (blue).

For fluorescent IdU/CldU staining, sections were treated with 0.01 M citrate buffer (pH 6.0) by microwaving for 6 min. Alexa Fluor anti-mouse-546 and anti-rat-488 (Thermo Fisher Scientific, Waltham, MA, USA, 1:200) were used as secondary antibodies. DAPI was used to visualize the nucleus. Fluorescent imaging was performed using a Keyence BZ-X710 microscope (Keyence Corporation of America, Itasca, IL, USA) and a Leica TCS SP8 confocal microscope (Leica Biosystems, 35578 Wetzlar, Germany).

### 2.4. In Situ Hybridization

To generate RNA probes, PCR was performed using cDNA from embryonic day 8 chicken skin. PCR primers were as follows: Sox9 (forward, ctcaagggctacgactggac; backward, atgtccacgtctcggaaatc); LGR6 (forward, tgattacgccttccagaacc; backward, gctctaggacacggaggttg). PCR products were inserted into p-drive (Qiagen Inc., Germantown MD 20874 USA) to make antisense RNA probes. K15 probes were from Wu et al. (2018) [35]. Section in situ hybridizations were performed according to procedures described in [35]. Diluted eosin was used for faint counterstaining.

### 2.5. Statistical Analysis

In Figure 3L, the data are mean ± SD. All comparisons were made by applying a paired two-tailed Student’s *t*-test.

## 3. Results

### 3.1. Comparison of Feather Regeneration Between Plucking During the Resting Phase or Growth Phase

**Figure 2:**
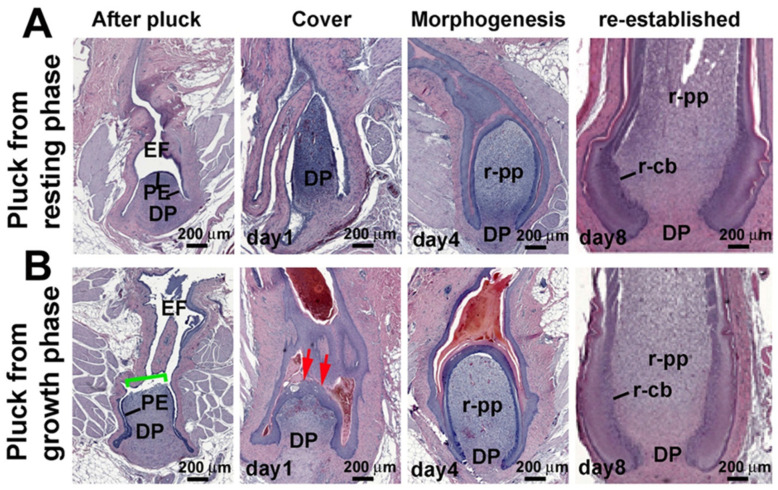
**Comparison of the regenerative process of feather follicles after plucking in the resting and growth phases.** Feathers can be regenerated after plucking in the resting phase or the growth phase. Both phases progress through the wound cover (day 1), morphogenesis (day 4), and re-established stages (day 8). In the re-established stage, feather branching starts, and the epidermal collar bulge forms. (**A**) Feather was plucked during the resting phase. The DP was surrounded by PE and was activated to regenerate. No re-epithelization events occurred. (**B**) Feathers were plucked during the growth phase. The PE was absent from the top of the DP (green bracket). The denuded DP quickly re-epithelialized and was covered by the epithelium (red arrows). DP, dermal papilla; EF, empty follicle; PE, papillary ectoderm; r-cb, regenerated collar bulge; r-PP, regenerated pulp.

To examine the feather regeneration process in the resting phase or growth phase, we plucked dorsal contour feathers from 1-year-old male White Leghorn chickens. Feather follicles were collected at 0 h (immediately after plucking), 1 day, 4 days, and 8 days. When feathers were plucked during the resting phase (Figure 2A), the PE covered the DP (Figure 2A, first column). The apical DP differentiated on day 1 and formed the regenerated pulp (r-pp) by day 4. On day 8, a new feather follicle was established containing a regenerated collar bulge (r-cb). In contrast, when feathers were plucked during the growth phase (Figure 2B), the top of the DP became denuded (green bracket), and re-epithelialization took place in 1–2 days (red arrows). By 4 days, feather morphogenesis had caught up with those plucked in the resting phase.

### 3.2. LRCs and TA Cell Activities During Feather Regeneration Following Feather Plucking in the Resting or Growth Phases

**Figure 3:**
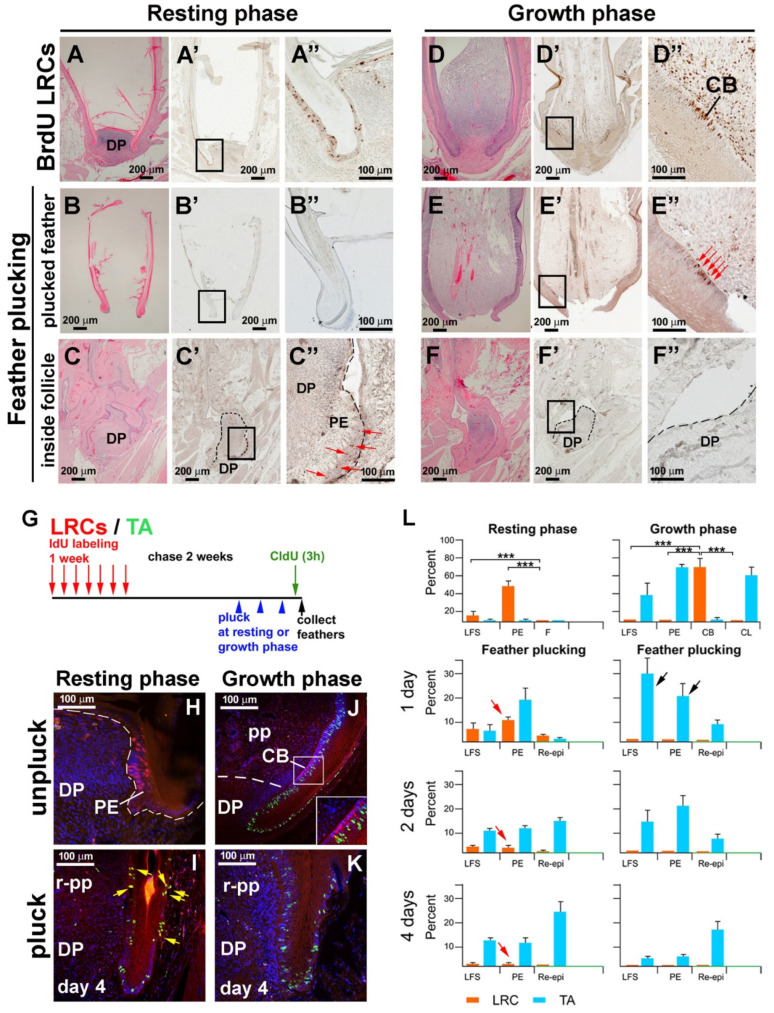
**Distribution of LRC and TA activities after plucking in the resting and growth phases.** (**A**–**F”**) LRCs were retained in follicles plucked during the resting phase. (**D**–**F”**) LRCs were not retained in follicles plucked during the growth phase. (**A**–**F**,**H**,**E**) Staining. (**A’**–**F’**) Low magnification view of BrdU staining. (**A”**–**F”**) High magnification view of boxed areas in panels. (**A’**–**F’**) LRCs of resting and growth stage feather follicles were labeled with BrdU for one week, followed by a 10-day chase period. Plucking removed the differentiated feather from the follicle. When plucking occurs in a resting stage follicle, the papillary ectoderm, which contains LRCs at this stage, remains in the follicle. When plucking occurs in a growth stage follicle, the collar bulge, which contains most LRCs at this stage, is removed, and the remaining follicle contains no LRC stem cells. (**G**) Feather LRC and TA cell labeling scheme. (**H**–**K**) LRCs (red) in both the resting- (**H**) and growth-phase ((**J**); inset, enlargement) unplucked follicles. Note that there were no TA cells (green) in resting-phase follicles. (**I**) Four days after the resting-phase follicle plucking, some cells became double-labeled (yellow arrows). (**K**) LRCs were absent in plucked growth phase follicles. TA cells (green) were abundant in the epidermis. (**L**) LRC and TA cell distribution in different regions of the progenitor niche. In the resting phase, LRCs were enriched in the PE and LFS. TA cells were absent. In the growth phase, LRCs were located in the collar bulge region, and TA cells were found in the collar region, papillary ectoderm, and lower follicle sheath. Notably, after feather plucking during the growth phase (black arrow), TA cell numbers were enriched in both the lower follicle and papillary ectoderm regions. The horizontal bar shows the significance of LRCs in the resting or growth phase (n = 3 feather follicles, *** *p* < 0.001, paired two-tailed Student’s *t*-test). CB, collar bulge; CL, collar; DP, dermal papilla; PE, papillary ectoderm; LFS, lower follicle sheath.

To study the location of the LRCs in the growth-phase and resting-phase feather follicles, we labeled the chicken with BrdU for 1 week and chased for 2 weeks. During this interval, proliferating cells diluted the BrdU label, but the label remained in slow-cycling LRCs. We found the LRCs can be retained in the PE in the resting phase (Figure 3A–A”) and in the collar bulge in the growth phase (Figure 3D–D”). When resting-phase feathers were plucked, LRCs were absent in the plucked feather shaft (Figure 3B–B”) but were present in the PE surrounding the DP (Figure 3C–C”). However, when the growth-phase feather was plucked, LRCs were lost in the plucked feather (Figure 3E–E”). The DP and PE were LRC-negative (Figure 3F–F”).

We next explored the difference in LRC/TA cell homeostasis in conditions with or without LRC stem cells. To visualize how feather LRCs and TA cells are reconfigured in response to injury, we double-labeled follicles with LRC and TA labeling, using IdU and CldU, respectively (Figure 3G). The resting phase follicle had numerous LRCs (red color) in the PE, whereas TA cells (green color) were seldom detected (Figure 3H). Following resting phase follicle plucking to simulate injury, LRCs in the PE and lower follicle sheath (LFS) were quickly activated, staining positive for both LRC and TA markers (yellow color) (Figure 3I, yellow arrows).

In the growth feather follicle, LRCs were enriched in the collar bulge (red color), and the TA cells were in the basal epidermal layer (green color) (Figure 3J). When we plucked growth phase feathers, completely removing the collar bulge, the active proliferating cells (green color) were seen in the lower follicle sheath epidermis, which thickened significantly (Figure 3K).

We further quantified the LRC and TA cell distribution in different regions of the progenitor niche (Figure 3L). In the resting phase, LRCs were enriched in the PE and LFS. The feather shaft was keratinized and was absent of LRCs. TA cells were absent. The number of LRCs gradually decreased in the regenerating process (red arrows). In the growth phase, LRCs localized to the CB, while TA cells localized to the CL, PE, and LFS. After plucking during the growth phase, TA cells were enriched in both the LFS and PE (black arrow). This result suggests that after plucking resting phase feathers, LRCs proliferated and took part in the blastema formation. In contrast, plucked growth phase follicles lacked LRCs, suggesting TA cells from both the PE and LFS were needed for repair and then regeneration.

### 3.3. Altered Molecular Expression Following Feather Plucking in the Resting or Growth Phases

**Figure 4:**
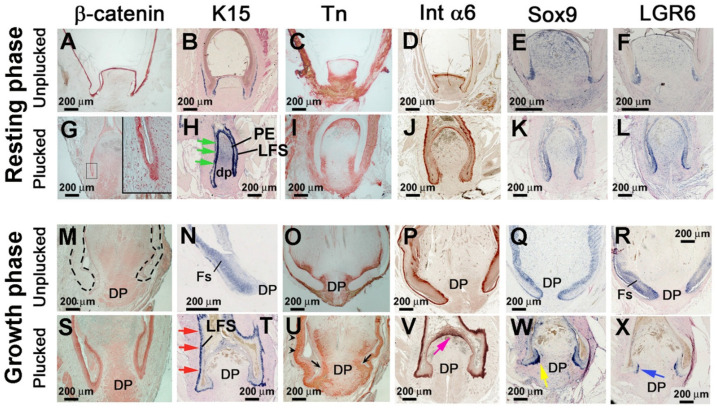
**Induced molecular expression in regenerating feather follicles after plucking in the resting and growth phases.** (**A**–**X**) Normal and plucked follicles were examined by immunostaining for β-catenin, tenascin C (Tn), and integrin-α6 (Int α6) and by in situ hybridization for Keratin 15 (K15), Sox9, and LGR6. (**A**–**F**) Normal resting phase feather follicle. (**G**–**L**) Feather follicles 1 day after plucking from resting phase. Inset in G shows nuclear β-catenin-positive cells in the PE. Green arrows in H indicate the increased K15 in the PE. (**M**–**R**) Normal growth-phase feather follicle. (**S**–**X**) Feather follicles 1 day after plucking during the growth phase. Red arrows in T indicate increased K15 expression in the LFS. Black arrow and arrowhead in U indicate expanded Tn expression in the LFS or DP, respectively. The pink arrow in (**V**) indicates increased integrin-α6 in the re-epithelization region. Yellow and blue arrows in (**W**,**X**) indicate the localized expression of Sox9 and LGR6, respectively, in the PE. DP, dermal papilla; FS, feather sheath; LFS, lower follicle sheath; PE, papillary ectoderm.

To identify molecules required for early regenerative stages, we compared molecular changes that accompany repair versus regeneration. Immunostaining and in situ hybridization were used to screen for molecules induced by feather plucking (Figure 4). We found that stem-cell-related genes (β-catenin, KRT15, integrin-α6, Sox9, and LGR6) are enriched in the resting-phase PE (Figure 4A,B,D–F). KRT15, integrin-α6, Sox9, and LGR6 are hair stem cells markers, but they identify different stem cell subpopulations in the hair follicle and have distinct roles [36,37,38,39]. KRT15 and Sox9 are mainly expressed in the bulge (a reservoir for quiescent hair follicle stem cells). Integrin-α6 is a general marker for stem cells and is broadly expressed in the basal layer and hair follicle. LGR6 marks highly multipotent stem cells capable of contributing to multiple skin lineages.

When feathers were plucked in the resting phase, nuclear staining β-catenin is evident, suggesting that Wnt/β-catenin signaling pathway activation is important in the regeneration process (Figure 4G). KRT15, Sox9, and LGR6 expression is enhanced in the PE (Figure 4H,K,L), whereas integrin-α6 is enhanced in the LFS (Figure 4J). Tenascin C, associated with regeneration and new growth [40], is present in normal resting-phase DP and dermal cells surrounding the feather follicle (Figure 4C). In the resting-phase plucked follicle, tenascin C expression is enriched in the peripheral DP (Figure 4I).

In contrast, in the growth phase plucked follicle, we did not find β-catenin nuclear staining (Figure 4S, compared to 4G). However, we observed strong expression of KRT15 in the LFS (Figure 4T, red arrows), increased expression of integrin-α6 on top of the re-epithelialized DP (Figure 4V, pink arrow), and localized Sox9 and LGR6 expression in the base of the blastema (Figure 4W,X, yellow and blue arrows). In addition, tenascin C shows increased expression in the follicle mesenchyme in the LFS (arrow heads) and the peripheral DP (black arrow) (Figure 4U). These data show a distinct molecular profile in the growth phase plucked follicle wound-repair process. Based on these data, molecules associated with stem cells and regeneration show dynamic expression in different follicle regeneration scenarios, implying that there are different ways to rebuild the stem-cell niche.

### 3.4. The Lost Regeneration Ability After Removing the Epithelial and Dermal Components of the Lower Follicle Can Be Restored Through DP Transplantation

**Figure 5:**
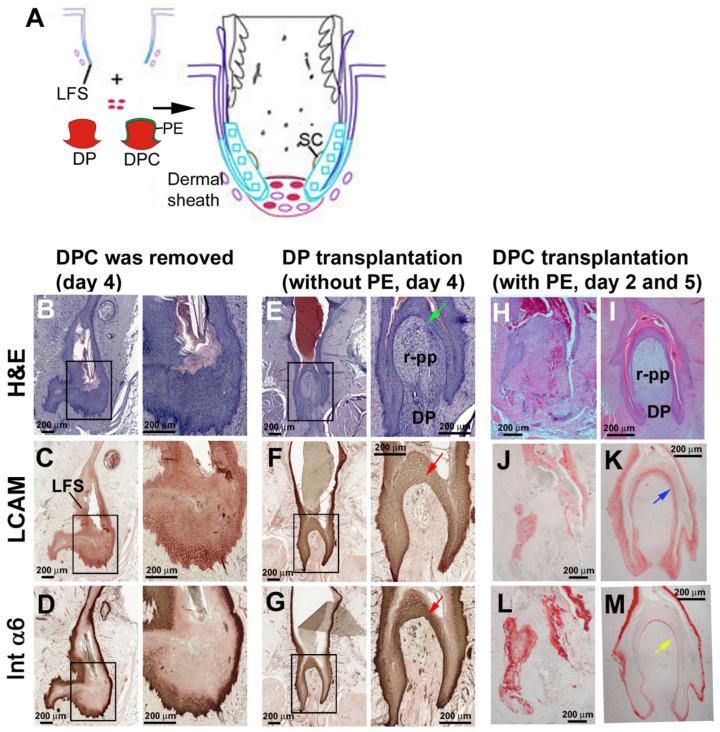
**Feathers failed to regenerate after follicle base removal but regained regenerative abilities after DP or DPC transplantation.** (**A**) Cartoon depicting chimeric follicle re-establishment. Both DC and DPC transplantation can generate a full-length feather. (**B**–**D**) A resting-phase feather follicle 4 days after the DPC was removed. Epithelial cells healed without feather follicle architecture. (**E**–**G**) A feather follicle 4 days after DP (DP without PE) transplantation. The DP induced blastema formation. Note that the newly formed epidermis covered the DP (green arrow). Since the transplanted DC did not have PE, the epidermis was from the LFS of the recipient follicle. LCAM and integrin-α6 (Int α6) were positive in multiple cell layers of this newly formed epidermal layer (red arrow). The boxes in the left panels indicate the magnified areas shown in the right of panels of **B**–**G**. (**H**–**M**) DPC (DP with PE) transplantation. Note that LCAM was positive in the epidermal layers close to the newly formed pulp (blue arrow), whereas integrin-α6 was positive in the basal cells of the feather epidermis (yellow arrow). DP, dermal papilla (without PE); DPC, dermal papilla complex (with PE); LFS, lower follicle sheath; r-PP, regenerated pulp; SC, stem cells.

Classical tissue recombination experiments used DP transplants to study tissue interactions [41]. We used this approach to re-evaluate the origin of cells needed to rebuild chimeric follicles (Figure 1A, right lower loop). To avoid excessive injury, resting follicles were often used for transplantation. Here we transplanted the DP to the bottom of the resting stage follicle to evaluate its regenerative ability. Transplantation was performed using two conditions. One condition involved the DP without the PE. The PE is tightly attached to the resting-phase DP and must be carefully removed after treatment with 2X CMF (see methods). The other condition involved the DP with the PE, which we call the dermal papilla complex (DPC) (Figure 5A).

When the DPC was removed from a host follicle, without transplanting a new DP, the host follicle epithelia proliferated and closed the wound but did not form feathers (Figure 5B). Liver cell adhesion molecule (LCAM, or E-Cadherin) stained the follicle sheath (Figure 5C), whereas integrin-α6 staining highlighted the basal layer follicle sheath cells (Figure 5D).

After transplanting the DP (without PE) to the DPC excised host follicle, the DP and regenerated pulp became encased by epidermal cells (Figure 5E, green arrow). These newly formed epidermal cells were LCAM and integrin-α6 positive (Figure 5F,G), indicating that they originated from the recipient follicle LFS.

In comparison, when the DPC (DP and PE) was transplanted to the DPC-removed host follicle, we observed a mixing of host and recipient epidermal cells on day 2 (Figure 5H). LCAM and integrin-α6 staining were not continuous at this stage (Figure 5J,L), implying that the donor PE and de-differentiated host lower follicle sheath cells had mixed to rebuild new feather epidermis. By day 5, the follicle structure had been established, with clear DP and pulp surrounded by continuous epidermis. LCAM stained the follicle sheath and feather epidermis (Figure 5K, blue arrow), whereas integrin-α6 stained the basal layer cells in follicle sheath and feather epidermis (Figure 5D, yellow arrow). This result suggests that DP cells can induce the follicle sheath epidermal cells to migrate and cover the nude DP, thus re-establishing the blastema for feather regeneration.

## 4. Discussion

### 4.1. Collar Bulge and PE Stem Cells Represent Residential Stem Cells in the Growth and Resting Phases, Respectively, While Dermal Sheath Epithelium Are Induced by DP to De-Differentiate and Regenerate New Stem Cells

**Figure 6:**
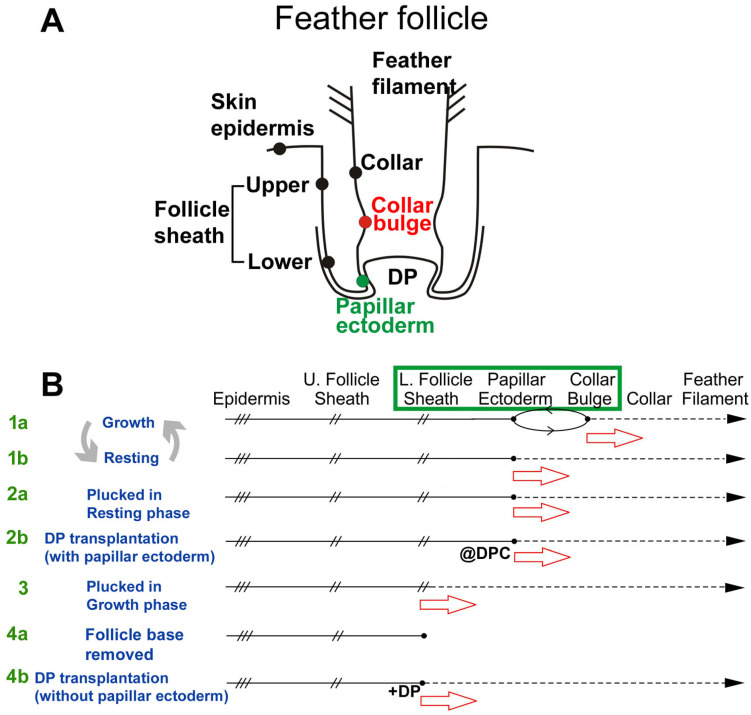
**Re-establishing a regenerative field after the loss of feather stem cells.** (**A**) Schematic drawing of a feather follicle. Major cellular states along the follicle wall are shown in a linear fashion. Each black dot represents different epidermal cell populations. (**B**) The progenitor population responds to regeneration, using different lines of response. (1a, 1b) Physiological cycling, using collar bulge and papillary ectoderm, respectively. (2a) Feather plucked in the resting phase. Papillary ectoderm is used. (2b) DP transplantation experiment [44]. Papillary ectoderm is used. @ indicates recombination using DPC. (3) Feather plucked in the growth phase. Lower dermal sheath cells are activated to regenerate. (4a) Follicle base removed. No regeneration. (4b) Transplantation of DP to condition 4a can restore regenerative follicles. + means added. The green box indicates the progenitor niche region. Cells with higher stem cell potential flow toward the TA and differentiated niche, but this is adjusted in response to damage. The red arrow indicates cellular flow.

The feather follicle consists of an expanded niche composed of LRCs, TA cells, and differentiated cells (feather sheath) that have latent stem cell potential (Figure 6A) [22,25,26]. Normally, cells form LRCs and TA sub-populations in specific niche locations and follow directional cellular flow (Figure 1C,C’). During physiological cycling, LRC stem cell clusters ascend and descend between the collar bulge and PE regions in the growth and resting phases, respectively.

In the resting phase, the follicle base includes the PE (epidermal stem cells) and DP (dermal signaling center). We showed that when the follicle base is excised, no feathers can regenerate. With a transplanted DP, host cells mix with donor cells to re-establish a new regenerative wound field, capable of generating fully functional feathers. These results suggest that the DP maintains bulge stem cell properties. Therefore, stem cells represent a functional state; cells are only conferred with stem cell properties when they are next to the proper stem cell niche.

While there is a hierarchy of progenitor cells during normal feather cycling, they are flexible and can replenish stem cells when stem cells are lost. According to this hierarchy, feather progenitors can switch between LRC and TA populations dynamically to cope with various forms of injuries, repairing or regenerating the spatial organization until stem cell homeostasis is re-established (Figure 6B). The results here are consistent with the perspective that stem cell behavior can exhibit dynamics like a flowing river [42] to replenish lost cells at the injured location.

### 4.2. Regenerative Strategy Learned from Wounded Feather Follicles

The regenerative strategy here implies that residential stem cells, including the collar bulge or PE, can be activated stepwise to participate in feather regeneration. First, normally, the collar bulge stem cells are used to generate feather filaments; however, when the collar bulge is not available, PE stem cells are activated to rebuild the collar bulge, which then generates feather filaments. When both epithelial stem cell components are lost, the lower feather sheath epithelium can be activated to re-epithelize the denuded DP. Together, they form a blastema and regenerate stem cells to restore feather filament formation. However, regeneration cannot recur without either a portion of the original DP or a transplanted DP.

Similar stepwise regenerative strategies may also be used in other organs. Some regenerative organs having a less distinct niche architecture may obscure interactions between epithelial stem cells and their dermal niche. Identifying the factors that regulate these processes will enhance our ability to elicit a regenerative response after wounding. We can apply a similar concept to feathers when examining hair regeneration. In hair cycling, the bulge contains stem cells. In response to severe wounds, bulge stem cells can be redirected to support re-epithelialization of the epidermis. Following bulge stem cell apoptosis after hair plucking, hair germ cells and the infundibulum can revert to form new bulge stem cells [43].

## 5. Conclusions and Future Directions

Classical tissue recombination experiments produced some controversy [15], and we speculate this may be due to the incomplete separation of the DP and PE, and the competition between host and donor cells forming the chimeric follicles. We need to characterize the molecular events involved. In the aspect of epidermis, follicular epidermal cells must be competent to de-differentiate into feather stem cells. There might be a difference between the upper follicle sheath and the lower follicle sheath. We hypothesize that the epigenetic profiles of collar bulge stem cells, PE stem cells, the lower feather sheath, and the upper feather sheath are different, underlining their different abilities to respond to wounding. We hope to delve into these differences in future studies. In the aspect of the dermal side, dermal fibroblasts are not sufficient to induce lower follicle sheath de-differentiation, but the DP is. We would like to learn which molecular pathways are involved. With a clearer understanding of wound healing and the blastema regeneration processes, we hope to uncover fundamental principles and candidate signaling pathways that can be translated to advance in organ regeneration.

## 6. Limitations of the Study

While the feather follicle provides distinct morphology and a larger size for experimentation, its resolution is limited by the lack of genetic tools available in transgenic mice. Ideally, a lineage-tracing tool using transgenic chickens with a reporter system similar to ROSA26 would enable precise tracking of stem cell dynamics. By employing cell type-specific Cre drivers, we could assess the observed strategy of stem cell regeneration. Furthermore, similar technology would facilitate the generation of specific cell clusters labeled with fluorescent markers, allowing for their isolation by cell sorting and molecular characterization. Although transgenic chickens have been produced, the process remains inefficient. To address this, we are collaborating with other researchers to develop more robust methodologies.

## Data Availability

Original data are available upon request.

## Abbreviations

The following abbreviations are used in this manuscript:

CB	collar bulge
CL	collar
EF	empty follicle
DP	dermal papilla
DPC	dermal papilla complex
LRC	label-retaining cells
PE	papillary ectoderm
LFS	lower follicle sheath
PP	pulp
r-cb	regenerated collar bulge
r-PP	regenerated pulp
SC	stem cells
TA cells	transient amplifying cells
UFS	upper follicle sheath
